# Die außen- und sicherheitspolitischen Positionen der SPD: Sozialdemokratische Außenpolitik ist multilaterale Außenpolitik

**DOI:** 10.1007/s12399-021-00849-6

**Published:** 2021-05-19

**Authors:** Gabriela Heinrich

**Affiliations:** Fraktion der SPD im Deutschen Bundestag, Platz der Republik 1, 11011 Berlin, Deutschland

**Keywords:** SPD, Deutsche Außenpolitik, Multilateralismus, EU, Rüstungskontrolle, SPD, German foreign policy, Multilateralism, EU, Arms control

## Abstract

Die internationale Ordnung ist in den vergangenen Jahren zunehmend volatil geworden. Bereits vorliegende Trends – Populismus, Angriffe auf die multilaterale Ordnung, Digitalisierung und Automatisierung, die Klimakrise – haben sich teils dramatisch verschärft. In dieser zunehmend unsteten Welt ist ein souveränes Europa mehr denn je Dreh- und Angelpunkt der Rolle Deutschlands in der Welt. In diesem Rahmen können wir den Multilateralismus stärken, die transatlantische Partnerschaft weiterentwickeln und neuen Herausforderungen (digitale Souveränität, aufstrebende Mächte) begegnen.

## Abstract (en)

The international order has become increasingly volatile. Already existing trends - populism, attacks on the multilateral order, digitalisation and automation, the climate crisis - have intensified. In this increasingly volatile world, a sovereign Europe is more than ever the cornerstone of Germany’s role in the world. Within this framework, we can strengthen multilateralism, further develop the transatlantic partnership and meet new challenges (digital sovereignty, emerging powers).

## Einführung

Die Jahre der jetzt zu Ende gehenden Legislaturperiode waren Zeiten dramatischer Umbrüche in der internationalen Politik. Darunter fällt die Corona-Pandemie, ein Ereignis von historischer Tragweite, dessen gesundheitliche, ökonomische, soziale, aber auch politische Folgen die Welt für Jahre und vermutlich sogar Jahrzehnte prägen werden. Zugleich haben sich viele langfristige Trends und Herausforderungen dramatischer als bisher manifestiert – vom Klimawandel, über den durch Digitalisierung und Automatisierung verursachten Strukturwandel, bis hin zur Rolle des Populismus und seiner Auswirkungen auf die internationale Politik.

Aus deutscher und europäischer Sicht hatte die Präsidentschaft Donald Trumps, seine offen zur Schau getragenen Verachtung für multilaterales, regelgebundenes Handeln und internationale Organisationen sowie sein Hofieren von Diktatoren und Populisten einen besonders dramatischen Effekt. Auch wenn nach nur einer Amtszeit mit Joe Biden nun ein Verfechter von Diplomatie und multilateralen Bemühungen ins Weiße Haus eingezogen ist, werden die Trumpschen Chaosjahre Spuren hinterlassen. Gerade für uns Europäer waren die vergangenen Jahre in dieser Hinsicht eine Herausforderung, die bisherige Gewissheiten im transatlantischen Verhältnis in Frage gestellt hat.

Auch deshalb ist in einer zunehmend volatilen Welt ein souveränes Europa für die Sozialdemokratische Partei Deutschlands (SPD) mehr denn je Dreh- und Angelpunkt der Rolle Deutschlands in der Welt. Es ist nur folgerichtig, dass die Stärkung Europas einer der drei großen Schwerpunkte des Kanzlerkandidaten Olaf Scholz ist. Wir wollen in der nächsten Legislaturperiode die europapolitische Dynamik, die sich 2020 entfaltet hat, als Motivation nutzen und an den durchaus vorhandenen positiven Entwicklungen des Krisenjahrs anknüpfen, denn: Die Corona-Pandemie hat gezeigt, dass die Staaten der Europäischen Union im Angesicht großer Herausforderungen handlungsfähig sind. Mit dem europäischen Wiederaufbauprogramm ist ein wichtiger Integrationsschritt gelungen. Das Programm, besonders die Beschlüsse zu einer besseren Eigenmittelversorgung der EU und zur Vergabe gemeinsamer Anleihen im großen Stil, stellt die Keimzelle einer echten Fiskalunion und somit einer souveräneren, handlungsfähigeren EU dar.

Ein geeintes und starkes Auftreten Europas in der internationalen Politik ist wichtiger als je zuvor. Die friedenspolitischen Herausforderungen nehmen zu – und sie sind vielfältig: Gesundheitskrise, Klimawandel und Ungerechtigkeit verschärfen bestehende Konflikte und entfachen neue. Autonome Waffensysteme senken die Schwelle für kriegerische Handlungen. Kernwaffen erleben ein Comeback, während es kaum noch international verbindliche Vereinbarungen über die Kontrolle und die Abrüstung von Massenvernichtungswaffen gibt. Digitaler Fortschritt macht uns verwundbar für Cyberangriffe.

## Priorität für multilaterale Zusammenarbeit

Die Covid-19-Pandemie unterstreicht einmal mehr die Notwendigkeit gemeinsamen multilateralen Handelns. Natürlich gibt es Herausforderungen, die die Nationalstaaten besser selbst lösen können. Pandemien, globale Wirtschafts‑, Finanz- und Entwicklungskrisen sowie die Erderwärmung gehören jedoch nicht dazu. Zur Bewältigung solcher Herausforderungen müssen multilaterale Institutionen gestärkt, aber nicht überfrachtet werden. Ohne Zweifel ächzen viele multilaterale Institutionen bereits jetzt unter der Last ihrer Aufgaben. Viele müssen dringend reformiert werden.

Internationale Organisationen können jedoch nur dann richtig funktionieren, wenn sich die Staaten über ihre Bedeutung einig sind und die Voraussetzungen schaffen, dass die Institutionen auch handlungsfähig sind. Sie sind maßgeblich vom Entschluss‑, Entscheidungs- und Finanzierungswillen ihrer Mitgliedstaaten abhängig. Die Missachtung und Unterausstattung wichtiger multilateraler Institutionen haben multilaterales Handeln fragmentiert und teilweise blockiert. Politische Unterstützung ist deshalb dringend geboten. Ein wichtiger Schritt hierfür ist die Allianz für Multilateralismus, die Bundesaußenminister Heiko Maas mit seinem französischen Amtskollegen und vielen weiteren Staaten ins Leben gerufen hat (Auswärtiges Amt 2019). Sie ist genau zum richtigen Zeitpunkt gekommen.

Insbesondere die Vereinten Nationen brauchen Unterstützung um ihren Auftrag der Friedenssicherung und der Wahrung der Menschenrechte zu erfüllen. Reformen sind hierfür dringend notwendig. Diese sollten wir vorantreiben, indem wir die Reforminitiativen des UN-Generalsekretärs unterstützen. Dazu gehört auch eine faire Lastenverteilung zwischen unseren europäischen und transatlantischen Verbündeten wie auch unseren Partnern in der Welt. Dies sollte sich aber auch in einem ständigen europäischen Sitz und einer angemessenen Repräsentanz des Globalen Südens im Sicherheitsrat der Vereinten Nationen widerspiegeln.

Auch das internationale Menschenrechtssystem braucht dringende Unterstützung. Die Unteilbarkeit und universelle Geltung der Menschenrechte ist für uns Sozialdemokrat*innen nicht verhandelbar. Menschenrechte sowie das humanitäre Völkerrecht sind die Richtschnur unseres Handelns. Wir sehen aber, dass eine Reihe von Staaten Menschenrechte verletzen und gezielt das internationale Menschenrechtssystem schwächen. Um Menschenrechte durchzusetzen, müssen wir diejenigen schützen, die für sie eintreten. Es ist gut, dass im EU-Rahmen unter deutscher Ratspräsidentschaft ein Menschenrechtssanktionsregime geschaffen wurde (Europäische Kommission 2020a). Dies muss nun konsequent genutzt werden. Dazu gehören Einreiseverbote und das Einfrieren von Konten. Auch die Möglichkeiten der weltweiten Strafverfolgung von Menschenrechtsverletzer*innen auf Grundlage des im deutschen Strafrecht verankerten Weltrechtsprinzips sollte mit diesem Ziel weiter gefördert und der Internationale Strafgerichtshof gestärkt werden.

## Frieden sichern durch Diplomatie und zivile Krisenprävention

In den vergangenen zwei Jahrzehnten hat die SPD maßgeblich die Ansätze von ziviler Krisenprävention und Konfliktbearbeitung geprägt. Mit dem friedenspolitischen Leitbild im Strategiedokument *Krisen verhindern, Konflikte bewältigen, Frieden fördern* (Auswärtiges Amt 2017) wurden Standards gesetzt, die weltweit geschätzt werden. Die Bundesrepublik Deutschland ist an der Spitze der Länder, die in Frieden investieren. Dies sollte weiter ausgebaut werden.

Bei der Entschärfung internationaler Krisen und der Vermittlung von Frieden nimmt Deutschland schon jetzt eine weltweite Führungsrolle ein. Das wollen wir Sozialdemokrat*innen weiter ausbauen, indem wir das Zentrum für internationale Friedenseinsätze (ZIF) stärken und ein hochprofessionelles Team von Friedensemissären für das Führen von Verhandlungen aufbauen. Friedensprozesse sind nur dann nachhaltig, wenn die Belange und Interessen von Frauen dort stärker berücksichtigt und wenn sie an Aushandlungsprozessen auch beteiligt werden. Deshalb wollen wir, dass die Resolution 1325 Frauen, Frieden, Sicherheit konsequent umgesetzt und weiterentwickelt wird. Um dies zu unterstützen, brauchen wir ein gendersensibles Frühwarnsystem und mehr genderorientierte Forschung zu Außen- und Friedenspolitik. Es gilt auf allen Ebenen der Anti-Genderbewegung entgegenzutreten, die international durch rückwärtsgewandte Kräfte stark zunimmt.

## Die nukleare Bedrohung hat sich weiterentwickelt und erfordert neue Antworten

Eine Welt ohne Atomwaffen ist und bleibt das Ziel sozialdemokratischer Außenpolitik. Entgegen der öffentlichen Wahrnehmung ist dieses Thema hochaktuell. Die Entwicklung neuer Generationen von Atomwaffen, die Weiterentwicklung von Nukleardoktrinen und die zunehmend seegestützte Stationierung von Nuklearwaffen erhöhen den Handlungsdruck. Hinzu kommt, dass weltweit Spannungen zunehmen, das Risiko einer Eskalation ist also durchaus gegeben. Die Denuklearisierung Nordkoreas stagniert, das Nuklearabkommen mit dem Iran droht zu scheitern.

Vor diesem Hintergrund hat die SPD die Mitgliedschaft Deutschlands im UN-Sicherheitsrat genutzt, das Thema nukleare Abrüstung wieder auf die internationale Tagesordnung zu setzen (Auswärtiges Amt 2020). Im Rahmen der Stockholm-Initiative für nukleare Abrüstung wurden zahlreiche konkrete Vorschläge vorgelegt. Wir setzen uns für eine abrüstungspolitische Offensive ein. Dazu gehört, dass bestehende Vereinbarungen über Rüstungskontrolle und Abrüstung, vorrangig der New START- und der Open Skies-Vertrag, unbedingt gerettet sowie die Verpflichtungen aus dem Nuklearen Nichtverbreitungsvertrag umgesetzt werden müssen. Die Vereinbarung der neuen US-amerikanischen Regierung mit der russischen Führung über eine Verlängerung des New START-Vertrags um fünf Jahre macht hier Hoffnung. Sie schafft hoffentlich auch den Raum, neue Abrüstungsinitiativen zu entwickeln. Ein Moratorium und Verhandlungen zwischen den USA und Russland zur verifizierbaren, vollständigen Abrüstung im substrategischen Bereich mit dem Ziel, die in Europa und in Deutschland stationierten Atomwaffen endlich abzuziehen und zu vernichten, muss der nächste Schritt sein.

Zudem bringt der im Rahmen der Vereinten Nationen beschlossene Atomwaffenverbotsvertrag eine weitere Dynamik in die Bemühungen für eine nuklearwaffenfreie Welt. Aufgrund seiner Bündnisverpflichtungen kann Deutschland dem Vertrag nicht beitreten. Es sollte sich aber als Beobachter bei der Vertragsstaatenkonferenz des Atomwaffenverbotsvertrags einbringen und so die Intentionen des Vertrages konstruktiv begleiten.

Gemeinsam mit der neuen US-Administration gilt es nun auch die Gespräche wieder aufzunehmen, wie eine vollständige Umsetzung des internationalen Atomabkommens Joint Comprehensive Plan of Action (JCPOA) mit dem Iran erfolgen kann. Allerdings ist die Lage hier ungleich komplexer als im Jahr 2015, in dem das Abkommen ursprünglich geschlossen wurde. Auf iranischer Seite ist das Vertrauen in Verhandlungslösungen gering, die moderaten Kräfte sind durch das Ausbleiben von wirtschaftlichen Erfolgen durch das JCPOA diskreditiert. Die regionale Sicherheitslage hat sich nicht entspannt, sondern in großen Teilen verschlechtert. Europa fällt bei der Deeskalation und der Vorbereitung eines neuen Nuklearabkommens eine entscheidende Mittlerrolle zu. Dabei sollte Deutschland im Kreise der E3 eine proaktive Führungsrolle ergreifen. Konkret hieße das, die USA möglichst bald zu spürbaren Sanktionserleichterungen zu bewegen, wenn der Iran im Gegenzug zu den Bestimmungen des JCPOA zurückkehrt und insbesondere die Urananreicherung nachprüfbar in den vom JCPOA vorgesehenen Rahmen zurückführt.

Im 21. Jahrhundert wandelt sich die Technologie der Kriegsführung massiv. Deshalb muss Rüstungskontrolle auch in neuen Bereichen wie Biotechnologie, Cyber und Künstliche Intelligenz etabliert werden. Insbesondere die Ächtung autonomer tödlicher Waffensysteme bleibt auch künftig ein Ziel sozialdemokratischer Politik. Hierfür werden wir ein internationales Regelwerk miterarbeiten. Bei allen Bemühungen um Abrüstung muss stärker als bisher auch China einbezogen werden.

## Stärkung der Bundeswehr im Rahmen von NATO und europäischer Zusammenarbeit

Die NATO ist und bleibt ein tragender Pfeiler der transatlantischen Partnerschaft und für Europas Sicherheit unverzichtbar. Nicht erst das erratische Handeln Trumps hat aber gezeigt, dass die EU parallel dazu sicherheits- und verteidigungspolitisch eigenständiger werden und die Einsatzbereitschaft ihrer Streitkräfte unter neuen Herausforderungen modernisieren muss. Eine europäische Armee als Teil der Friedensmacht Europa ist das Ziel. Durch die Bündelung europäischer Rüstungskooperation könnten wir Synergien nutzen und unnötige Mehrausgaben einsparen.

Die Bundeswehr leistet als Parlamentsarmee einen verantwortungsvollen Beitrag zur kollektiven Sicherheit und Verteidigung. Dies beweist sie mit ihren vielfältigen Auslandseinsätzen. Auch für die medizinische und logistische Unterstützung bei der Eindämmung der Pandemie – auch über Grenzen hinweg – gebührt den Soldat*innen unser ausdrücklicher Dank. Für die SPD steht fest, dass wir nur mit einer gut ausgestatteten und modernen Bundeswehr unseren Aufgaben als zuverlässiger Partner auch in Europa und der NATO gerecht werden können. Die Erhöhungen der Investitionen im Verteidigungshaushalt in den letzten Jahren ist Ausdruck dieser Überzeugung. Die Soldat*innen verdienen die bestmögliche Ausrüstung und den höchsten Grad an Ausbildung. Auch mit Blick auf die Bewaffnung von Drohnen ist dies ein sehr wichtiges Argument. Jedoch hat nicht zuletzt der massive Einsatz von bewaffneten Drohnen als Angriffswaffen im Krieg zwischen Aserbaidschan und Armenien und im Libyenkonflikt neue grundlegende Fragen zur Bewaffnung von Drohnen aufgeworfen. Dies macht eine umfassende politische und gesellschaftliche Debatte dringend nötig, die dieser neuen Entwicklung in der praktischen Kriegsführung Rechnung trägt.

Mit Blick auf alle Rüstungsgüter bleibt für die SPD eine restriktive Rüstungsexportpolitik zentral. Die Ausfuhr deutscher Rüstungsgüter in Staaten außerhalb von EU-, NATO- und denen gleichgestellten Ländern wollen wir weiter einschränken, die Kontrolle über den endgültigen Verbleib der Waffen ausweiten und absolute Ausnahmen nur in streng begründeten Einzelfällen ermöglichen – öffentlich nachvollziehbar dokumentiert. Dafür braucht es auf nationaler Ebene ein Rüstungsexportgesetz und auf europäischer Ebene eine mit unseren Partnern abgestimmte Verschärfung der EU-Rüstungsexportvereinbarungen. Für uns ist klar: Jede Form der Rüstungskooperation mit Staaten, die weder Mitglied der EU noch der NATO sind, kann nur erfolgen, wenn diese den Vertrag über Waffenhandel ratifizieren und konsequent umsetzen.

## Ein starkes Europa als Kerninteresse unserer Politik

Im vergangenen Jahr jährte sich die Wiedervereinigung Deutschlands zum 30. Mal. In diesen 30 Jahren hat die Bundesrepublik Jahre des Wohlstands und des Friedens erleben können. Ermöglicht wurde dies unter anderem durch ein verlässliches multilateralejs Regelwerk, vor allem aber auch durch die Europäische Union. Die Einheit Europas ist unsere gemeinsame Chance auf eine bessere Zukunft im globalen 21. Jahrhundert. Nur durch Europa werden wir unsere Eigenständigkeit und unsere Art zu leben in einer multipolaren Welt verteidigen können. Hierfür müssen wir die Einheit Europas nach innen stärken und die EU gezielt dort weiterentwickeln, wo sie heute zu verletzlich ist und unserem Anspruch an Eigenständigkeit nicht genügt.

Mit der Bewältigung der Corona-Pandemie und ihren Folgen steht die EU vor einer der größten Herausforderungen seit ihrer Gründung. Wie schon bei der letzten großen Krise vor gut zehn Jahren bestand im Frühjahr und Sommer 2020 die akute Gefahr, dass sich ökonomische und soziale Spaltungen vergrößern, dass Misstrauen zwischen unseren Ländern entsteht und daraus folgender Populismus und Nationalismus das europäische Einigungswerk gefährdet. Unter Führung der SPD und ihrer Minister*innen sind wir einen anderen Weg gegangen: Ohne die SPD hätte es die historischen Entscheidungen zu einem gemeinsamen Wiederaufbaufonds nicht gegeben. Sozialdemokrat*innen haben die schädliche Politik der öffentlich verkündeten roten Linien beendet und Europa gerade auch während der deutschen EU-Ratspräsidentschaft im zweiten Halbjahr 2020 vorangebracht.

Darauf muss nun weiter aufgebaut werden. Innere Handlungsblockaden der EU müssen weiter abgebaut und die äußere Handlungsautonomie fortentwickelt werden. Die Europäische Union weiterzuentwickeln bedeutet aber auch bei jeder Übertragung von Aufgaben die vorhandenen Kapazitäten kritisch zu hinterfragen und wo nötig zu stärken. Dies ist auch eine Lehre aus der gemeinsamen Impfstoffbeschaffung.

Europa braucht nach Corona einen Neuanfang und den dringend nötigen Fortschritt bei der sozial-ökologischen Transformation in die Zukunft. Die Agenda 2030 der Vereinten Nationen mit ihrer globalen Nachhaltigkeitsstrategie ist für uns Richtschnur und zentrales Entwicklungsmodell, auf das wir hinarbeiten. Nur mit einer solidarischen und souveränen EU sind wir in der Lage, die Welt von morgen mitzugestalten und unserer Vision einer demokratischen, gerechten und nachhaltigen Gesellschaft näher zu kommen. Gestützt auf ein robustes Fundament gemeinsamer Grundwerte von Demokratie, Freiheit und Rechtsstaatlichkeit wollen wir die wirtschaftliche und soziale Stabilität der gesamten EU festigen. Ein solches Europa kann seinen Einfluss gleichermaßen zum Schutz und zur Stärkung europäischer Werte und Interessen einbringen, als selbstbewusste Friedensmacht auftreten und so eine kooperative, multilaterale Weltordnung mitgestalten.

## Die Europäische Union als selbstbewusste Friedensmacht

Europa muss außen- und sicherheitspolitisch souveräner, selbstbewusster und handlungsfähiger werden. Denn das ist die Voraussetzung, die europäischen Werte und Interessen in der Welt auch zukünftig zu wahren und nicht Spielball anderer Großmächte zu werden. Für ein souveränes Europa steht der Schutz seiner Bürger*innen und die Wahrung des internationalen Friedens an vorderster Stelle. Bereits heute ist die EU ein globales Schwergewicht im Bereich der *Soft Power*. Die SPD setzt sich dafür ein, dass Europa eine Vorreiterrolle bei internationaler Krisenprävention, Friedens- und Demokratieförderung sowie zum Schutz von Menschenrechten einnimmt. Zusammen mit ihren Mitgliedsstaaten ist die EU weltweit die größte Geberin in der Entwicklungszusammenarbeit (Europäische Kommission 2020b). Die EU-Haushaltsmittel für Entwicklungszusammenarbeit und humanitäre Hilfe sollten weiter gestärkt werden und die Umsetzung der transformativen Agenda 2030 für Nachhaltige Entwicklung zum zentralen Kernanliegen auch in der EU-Außen- und Entwicklungspolitik gemacht werden. Dazu gehört auch eine kohärente Handels- und Agrarpolitik.

Mehr denn je ist die Nachbarschaft Europas im Süden wie im Osten durch Krisen und Fragilität sowie durch die wachsende Einflussnahme anderer Staaten geprägt. Diese Herausforderungen muss die EU durch eine konzeptionell neu ausgerichtete Europäische Entwicklungs- und Nachbarschaftspolitik aktiv angehen. Insbesondere die Länder des Westbalkans sollten wir weiter an die Europäische Union heranführen und integrieren. Europa und Afrika verbindet eine enge Partnerschaft auf Augenhöhe, die weiter vertieft werden sollte.

Mehr Eigenständigkeit setzt höhere Handlungsfähigkeit voraus. Grundlegend dafür ist die Einführung von Mehrheitsentscheiden in der Außenpolitik – statt des jetzigen Einstimmigkeitsprinzips. Auch das Amt des Hohen Vertreters der EU für Außen- und Sicherheitspolitik sollte langfristig in Richtung der Position eines EU-Außenministers weiterentwickelt werden.

Ein Europa, das geschlossen auftritt, trägt als Vorbild zur Belebung eines funktionierenden und kooperativen Multilateralismus bei. Gerade in Zeiten des Umbruchs sind wir auf internationale Vertrauensnetzwerke angewiesen. Die progressiven Kräfte weltweit müssen zusammenarbeiten und verhindern, dass Populisten und Nationalisten die Chancen auf eine bessere Zukunft zerstören.

## Die Transatlantischen Beziehungen neu denken

Mit der Wahl Joe Bidens zum 46. Präsidenten der Vereinigten Staaten von Amerika hat sich eine deutliche Mehrheit der Bürger*innen der USA für gesellschaftlichen Zusammenhalt und gegen eine Politik der Ausgrenzung entschieden. Der Gewinn der Wahl durch Joe Biden und Kamala Harris und ihre Mehrheiten in beiden Kammern bietet trotz aller schweren Hypotheken und gesellschaftlichen Verwerfungen, die Trump hinterlassen hat, für Deutschland und Europa nun die Chance eines Neustarts der transatlantischen Beziehungen (SPD-Bundestagsfraktion 2021).

Die Vereinigten Staaten sind weiterhin der wichtigste und engste außereuropäische Partner Deutschlands. Uns verbinden mit der ältesten Demokratie der Welt die gemeinsamen freiheitlichen, demokratischen Werte, das Bekenntnis zu Rechtsstaatlichkeit und Menschenrechten, ebenso wie die Marktwirtschaft als wirtschaftlicher Ordnungsrahmen. Sie alle bilden die Grundlagen einer regelbasierten, multilateralen Weltordnung, für die Deutschland, Europa und die USA gemeinsam in der Welt eintreten.

Nicht alle Differenzen im transatlantischen Verhältnis sind auf die Präsidentschaft Donald Trumps zurückzuführen. Schon in den Jahren vor Trump wurden grundsätzliche Veränderungen in den transatlantischen Beziehungen sichtbar. Beispiele hierfür sind die Diskussion über die Lastenteilung in der NATO, die stärkere Hinwendung der USA zum asiatisch-pazifischen Raum, die Verhandlungspause zur Transatlantischen Handels- und Investitionspartnerschaft oder – unter Präsident George W. Bush – der Irakkrieg.

Es ist umso wichtiger, gemeinsam mit der neuen US-Regierung die Weichen für die Zukunft neu zu stellen und die transatlantischen Beziehungen proaktiv zu gestalten. Deutschland und Europa müssen mit der neuen US-Regierung eine transatlantische Partnerschaft auf Augenhöhe anstreben und auch bei unterschiedlichen Positionen zu einem respektvollen, konstruktiven und vorausschauenden Dialog zurückkehren. Die Partnerschaft zwischen Europa und den USA, die auf gemeinsamen und demokratischen Werten beruht, sollte grundsätzlich gestärkt und die Zusammenarbeit bei Themen wie Klimaschutz, globaler Gesundheitspolitik, Handel, Abrüstung und Sicherheitsfragen wieder intensiviert werden.

## Konstruktiver Dialog mit China zur Gestaltung der internationalen Ordnung

Gemeinsam mit unseren transatlantischen Partnern sollten wir auch einen engen Austausch zu unserer Chinapolitik führen. Die wachsende Bedeutung Chinas bringt eine Vielzahl an Herausforderungen, aber auch eine Vielzahl von Chancen mit sich. Ziel muss eine gemeinsame europäische Position sein, die fest in der Wertegemeinschaft des Westens verortet ist, die die europäische Souveränität in einer regelbasierten multilateralen Ordnung stärkt, und konstruktive, offene und transparente Beziehungen zu diesem zentralen Akteur von Morgen vertieft.

Es ist die Tradition sozialdemokratischer Politik, nicht nur über, sondern auch mit China zu reden, und dabei konstruktiv-kritische Fragen der Kooperation und des Wettbewerbs zu behandeln. Ohne den Dialog mit China ist die Gestaltung der ökonomischen, ökologischen, sozialen und politischen Herausforderungen unserer Zeit kaum vorstellbar. Die drei Dimensionen Partnerschaft, Wettbewerb und Systemrivalität strukturieren und begrenzen diesen Dialog. Chinas Wille zur aktiveren Gestaltung der internationalen Ordnung eröffnet die Möglichkeit zur Vertiefung der Zusammenarbeit, um gemeinsame Interessen auf globaler Ebene zu fördern. Dies umfasst auch die Bemühungen um Abrüstung, in die China stärker einbezogen werden sollte.

Gleichzeitig nehmen Interessens- und Wertekonflikte zu. Es stehen zwei verschiedene Modelle im Wettbewerb: das westliche Modell eines demokratischen Rechtsstaats mit freier und sozialer Marktwirtschaft und das chinesische Modell eines autoritären Staatskapitalismus. China ist somit nicht nur ein Kooperationspartner, sondern zugleich ein wirtschaftlicher Konkurrent und ein ideologischer Systemrivale. Wertekonflikte bestehen vor allem in den Bereichen Freiheit, Menschenrechte, Demokratie und Rechtsstaatlichkeit. Letztendlich bestimmt die Systemkonkurrenz das Ausmaß, wie die Partnerschaft mit China konkret ausgestaltet werden kann und beeinflusst auch die Art und Weise des wirtschaftlichen Wettbewerbs mit China (SPD-Bundestagsfraktion 2020a).

## Kritischer Dialog und zielgerichtete Sanktionen mit Blick auf Russland

Deutschlands und Europas Beziehungen zu Russland sind in den letzten Jahren immer wieder geprägt von Rückschlägen. Ob die völkerrechtswidrige Annexion der Krim, die Unterstützung der Separatisten in der Ostukraine, Cyberangriffe auf den Deutschen Bundestag oder die Anwendung des international geächteten chemischen Kampfstoffes Nowitschok zur Ausschaltung innenpolitischer Gegner: Russland bricht regelmäßig internationales Recht. Dies muss geächtet werden, die europäische Sanktionspolitik ist deshalb folgerichtig. Klar ist aber auch: Dauerhaften Frieden in Europa kann es nicht gegen, sondern nur mit Russland geben. Basierend auf den Werten und Prinzipien der Organisation für Sicherheit und Zusammenarbeit in Europa (OSZE) sollte eine neue europäische Ostpolitik entwickelt werden, die den Fokus auf eine gemeinsame und kohärente EU-Politik gegenüber Russland legt.

Voraussetzung für den Abbau von Spannungen ist allerdings eine konstruktive Dialogbereitschaft seitens Russlands. Dazu zählt auch, dass der Weg zu einer friedlichen Lösung des Ukrainekonflikts und damit einhergehend die Beendigung der Sanktionen maßgeblich von der Umsetzung der Minsker Vereinbarungen abhängt.

Das Vorgehen der russischen Regierung gegen Alexei Nawalny und die vor diesem Hintergrund stattfindenden landesweiten Proteste sind Zeichen der Schwäche Putins. Die endemische Korruption schwächt die Legitimation seiner Regierung. Das Risiko von Übersprunghandlungen ist durchaus real, es bleibt deshalb umso wichtiger, die wenigen verbleibenden Gesprächskanäle offen zu halten. Auch wirtschaftliche Verflechtungen und Projekte wie Nordstream 2 sind Elemente dieses Dialogs. Es ist wichtig, sie zu erhalten. Wertvoll in den Beziehungen zu Russland sind aber gerade auch die zivilgesellschaftlichen Kontakte. Eine zweigleisige Strategie sollte deshalb neben Sanktionen gegen Menschenrechtsverletzungen zivilgesellschaftliche Stimmen stärken, beispielsweise durch Visaerleichterungen, vor allem für junge Menschen.

Gleiches gilt mit Blick auf die Menschen in Belarus und ihrem Streben nach Demokratie und Freiheit. Die Gewalt und Repression der Sicherheitskräfte sind auf das Schärfste zu verurteilen. Stattdessen braucht es die unverzügliche Freilassung aller politischen Gefangenen und demokratische Neuwahlen unter der Aufsicht der OSZE.

## Ökonomische Hebel der Außenpolitik nutzen

Wirtschaftspolitik ist kein klassisches Element der Außenpolitik. Dennoch wäre es falsch, in Zeiten einer global integrierten Wirtschaft und globaler Herausforderungen die ökonomischen Aspekte und Hebel von Außenpolitik zu vernachlässigen. Dies zeigt sich insbesondere in den folgenden vier Bereichen:

### Faire Lastenverteilung weltweit

Mit den Beschlüssen zum Wiederaufbaufonds haben sich die Staaten der EU auch zur Schaffung echter europäischer Eigenmittel bekannt. Drei dieser Elemente, die Besteuerung digitaler Großkonzerne, eine CO2-Grenzabgabe sowie neue Einnahmen aus dem Emissionshandel sind von besonderer Relevanz. Neben Einnahmen für die EU haben sie auch große Auswirkungen auf die Frage gerechter Besteuerung und den Umbau hin zu einer CO2-neutralen Wirtschaft. Diese Elemente werden ein hohes Maß an internationaler Koordinierung im Rahmen von G7, G20 und der Organisation für wirtschaftliche Zusammenarbeit und Entwicklung (OECD) erfordern. Gleichzeitig bieten sie die Möglichkeit, gemeinsam mit *like-minded* Staaten das multilaterale Steuer- und Wirtschaftssystem weiterzuentwickeln. Denn auch über die EU hinaus haben wir Sozialdemokrat*innen das Ziel, einen neuen globalen steuerpolitischen Konsens zu etablieren. Vermögende sollen in die Pflicht genommen werden, zur Finanzierung des Gemeinwohls beizutragen. Es geht uns um eine globale Wirtschafts- und Finanzpolitik, die den Anspruch hat, zu gestalten und die Bedürfnisse der Menschen wieder in den Mittelpunkt des Wirtschaftens zu stellen. Das UN Tax Committee sollte zu einer globalen Steuerkoordinationsstelle aufgewertet und das OECD Inclusive Framework on BEPS im internationalen Kampf gegen Gewinnverkürzung und Gewinnverlagerung unterstützt werden. Steueroasen sollen so trockengelegt werden und gerechte Steuersysteme unter angemessener Beteiligung auch der Eliten im Globalen Süden geschaffen werden.

### Nachhaltiges Wirtschaften und Bekämpfung des Klimawandels

Um Klimawandel, Artensterben und übermäßigem Rohstoffverbrauch entgegenzuwirken, muss sich die Art und Weise, wie wir leben, konsumieren und produzieren, grundlegend ändern. Zukunft zu gestalten bedeutet heute, sich der Biodiversitäts- und Klimakrise als übergeordneter Herausforderung unserer Zeit zu stellen und konsequent daran zu arbeiten, den nach uns folgenden Generationen einen ökologisch intakten Lebensraum zu erhalten. Europa soll zum ersten nachhaltigen und treibhausgasneutralen Kontinent werden, spätestens 2050. Dies ist auch eine Chance. Es gilt Europas Potentiale zu nutzen, um international eine Vorreiterrolle bei der Bekämpfung des Klimawandels einzunehmen. Dem Energiesektor kommt dabei eine Schlüsselrolle zu. Wir müssen den Anteil Erneuerbarer Energien enorm steigern, den Energiemix diversifizieren und bestehende Abhängigkeit von fossilen Energielieferungen abbauen. Der Wandel weg von fossilen Brennstoffen und Öl wird auch in der internationalen Politik massive Veränderungen und Machtverschiebungen mit sich bringen und hat das Potential, Europa unabhängiger von Ressourcenlieferanten zu machen. Der digitale und ökologische Wandel bietet zudem die Chance, durch die Förderung von Innovationen dauerhaft international wettbewerbsfähig zu bleiben und zukunftsfähige Arbeitsplätze zu schaffen. Mit Investitionen in Weiterbildung können wir gute Arbeit in Zukunftssektoren schaffen.

### Digitale Souveränität

Der europäische Wirtschaftraum muss robuster und leistungsfähiger werden, die EU sollte als weltweiter Technologieführer auch in Zukunftssektoren unabhängiger von Dritten sein. Dafür braucht es neben der Entwicklung gemeinsamer Märkte und Infrastrukturen auch die Diversifizierung von Handelspartnern, der Entwicklung und finanziellen Unterfütterung strategisch wichtiger Zukunftstechnologien und den Schutz von Schlüsselindustrien (SPD-Bundestagsfraktion 2020b). Um in der Wirtschaft des 21. Jahrhunderts bestehen zu können braucht Europa eigene digitale Fähigkeiten. Unsere digitale Infrastruktur muss vertrauenswürdig und zugleich sicher vor Angriffen von außen sein.

### Handelspolitik für gute Arbeit weltweit

Die Handelspolitik braucht mehr Fair Play. Deutschland ist wie kaum ein anderes Land auf offene Märkte und eine funktionsfähige sowie regelbasierte globale Wirtschaft angewiesen. Um diese zu erhalten, braucht es ein stabiles, faires und demokratisches Handelssystem. Das umfasst die Stärkung der Welthandelsorganisation. Die Ernennung von Ngozi Okonjo-Iweala als Generaldirektorin ist ein ermutigendes Zeichen für das Bekenntnis der internationalen Gemeinschaft zu dieser Institution. Der dadurch entstandene Schwung sollte nun genutzt werden, um das Regelwerk zu erweitern, die Nachhaltigkeitsziele der UN einzubinden sowie die Durchsetzungsmöglichkeiten zu verbessern.

Es ist gut, dass die SPD durchsetzen konnte, dass in allen Handels‑, Wirtschaftspartnerschafts- und Investitionsabkommen der EU zukünftig neben verbindlichen sozialen Standards, wie den Kernarbeitsnormen der International Labour Organization (ILO) sowie menschenrechtlichen und ökologischen Standards, auch konkrete Beschwerde- und Sanktionsmechanismen vereinbart werden sollen. Dies – wie auch die Einhaltung menschenrechtlicher Sorgfaltspflichten entlang globaler Lieferketten, wie sie in einem Lieferkettengesetz auf nationaler und europäischer Ebene festgeschrieben werden soll – dient auch dem Ziel der Schaffung von guter Arbeit weltweit. Auch die wirtschaftliche Globalisierung muss den Menschen dienen. Menschenrechte enden nicht an Grenzen und die Menschheitsherausforderung Klimawandel kann nicht national bekämpft werden. Eine positive, progressive Zukunftsvision umfasst deshalb immer auch die internationale Dimension.
